# Treatment of Traumatic Tetanus with Calabar Bean and Chloral Hydrat

**Published:** 1871-11

**Authors:** C. B. Gallaway

**Affiliations:** Canton, Miss.


### TREATMENT OF TRAUMATIC TETANUS WITH CALABAR BEAN AND CHLORAL HYDRAT.

BY C. B. GALLAWAY, M. D., OF CANTON, MISS.

On the 3d of March last, I was called to see Jno. Carter, (Colored) age 28 or 30 years. At the first glance, it was apparent that he was suffering from Tetanus; all the symptoms being well marked. On inquiry, I ascertained that ten days previous, he had stuck a nail in his foot—examining the wound, I found no unusual heat or swelling, and the wound apparently, almost well. His muscles were all, more or less, rigid, the abdominal muscles, especially, were to the touch as hard as a board. The slightest effort to change his position, would produce tetanic contractions. As the bowels were constipated, I gave him hydrag. cholo. mite, grs. x.; podophillin, grs. j., at a dose, ordered pulv. calabar bean, grs. ii., every two hours, forbade all visitors, and had him kept quiet. Saw him again in the evening—found his bowels had acted well. His condition in every other respect, about the same—directed the calabar bean continued every two hours, and 20 grs. chloral hydrat to be given at bed time, and if he did not sleep, to repeat the dose in two hours.

4th. Had spent a comfortable night—his muscles were not so rigid, and swallowed without difficulty, pulse 90. Gave him chloral, 10 grs., and directed it repeated every six hours, and the calabar bean continued every two hours.

5th. Found him much improved, no muscular contractions except when he attempted to change his position, and then only slight—pulse 85. Directed the calabar to be given every four hours, and the chloral, as before, every six.

6th. He was so much improved that I did not think it necessary to visit him again. Directed him to continue the bean every six hours, a day or two, and take the chloral at night.

8th. Was called again to see him, and to my great astonishment, found him in the same condition, in which I first saw him, on the 3d, if anything, the spasms were more frequent—pulse 100, bowels regular. I directed the calabar bean to be given every two hours, and the chloral, at night, to be repeated every two hours, until he slept.

9th. Found such a marked abatement in his symptoms, and his spasms so slight, that I directed the medicine given at longer intervals. This treatment was continued until the 5th, increasing the interval a little every day. His nourishment was milk and beef tea. He was now so much improved that he could sit up. It is worthy of remark, that his bowels continued to act well after I gave him the chloral and podophillin. I have recently seen calabar recommended for constipated bowels. The question may arise, in the mind of the reader, may not recovery, in this case, be attributed to the chloral. I am free to say, my reliance was upon the calabar bean, and that I used the chloral more for its hypnotic effect than for any healing property it may possess.

Since writing the above, I have recieved the October number (1871) of the American Journal of the Medical Sciences. On page 560 you will find the following, which is to the point:

"Of the ten cases, treated exclusively by chloral, seven were instances of traumatic tetanus, and only one of these recovered; two cases of idiopathic tetanus were thus treated, of these recovered, as, also, did a case of the disease occuring fifteen days after childbirth. My experience thus far, has led me, therefore, to conclude, that I was hardly justified in trusting to the chloral alone, in the treatment of severe cases of this disease; as a hypnotic it seemed invaluable, but we must, if possible, do something more than put our patients to sleep in the management of bad cases of tetanus, and although calabar bean possesses an influence infinitely short of being a specific in this disease, nevertheless the extract of physostigma, if judiciously employed, has, I think, a salutary influence over some cases of tetanus."

